# To Assess the Efficacy of Various Disinfection and Hemostasis Procedures in Providing Postoperative Pain Relief Following Pulpotomy in Cases of Symptomatic Irreversible Pulpitis

**DOI:** 10.7759/cureus.45163

**Published:** 2023-09-13

**Authors:** Savitri Dronamraju, Muzaamill M Baig, Swetha K, Bhaskar K Naik, Harshita Gupta, Shikha Singh

**Affiliations:** 1 Department of Conservative Dentistry and Endodontics, Malla Reddy Institute of Dental Sciences, Hyderabad, IND; 2 Department of Conservative Dentistry and Endodontics, Sri Balaji Dental College, Hyderabad, IND; 3 Department of Conservative Dentistry and Endodontics, Malla Reddy Dental College for Women, Hyderabad, IND; 4 Department of Conservative Dentistry and Endodontics, SVS (Sri Venkata Sai) Dental College, Mahabubnagar, IND; 5 Department of Conservative Dentistry and Endodontics, Dr. Jaydev Dental Clinic, Hyderabad, IND; 6 Department of Conservative Dentistry and Endodontics, People's Dental Academy, Bhopal, IND

**Keywords:** postoperative pain relief, hemostasis, disinfection, pulpitis, pulpotomy, irreversible, ktp, naocl

## Abstract

Aim: The purpose of this study is to assess the efficacy of various disinfection and hemostasis procedures in providing postoperative pain relief following pulpotomy in cases of symptomatic irreversible pulpitis.

Materials and methods: The data from a cohort of 50 patients who received treatment with sodium hypochlorite (NaOCl) and another cohort of 50 patients who received treatment with potassium titanyl phosphate (KTP) laser were subjected to analysis. The patients were provided a questionnaire to evaluate pain levels before and after surgery. The patients documented their levels of postoperative pain at specific time intervals, including the sixth hour, first day, second day, third day, and eighth day. This was done using a 100 mm visual analog pain scale, where a marking of 0 mm indicated no pain and a marking of 100 mm indicated the highest level of pain, reflecting the severity of the pain experienced.

Results: The pain score of group B was significantly lower than that of group A on the first day. While no notable disparity was detected among the groups during the remaining postoperative periods, it is worth noting that the KTP laser exhibited comparatively lower pain scores. In both groups, the initial pain score before surgery was found to be significantly higher than the pain scores recorded at all subsequent time intervals after surgery. Within group A, it was observed that the pain score during the sixth hour after the surgical procedure was notably greater compared to the pain scores recorded during all other time intervals following the operation. In group B, the pain score at the sixth hour exhibited a statistically significant increase compared to the pain scores observed on the third day and eighth day.

Conclusion: The KTP laser group exhibited a lower postoperative pain score in comparison to the NaOCl group. The utilization of KTP laser-assisted pulpotomy demonstrated enhanced efficacy in alleviating pain among individuals diagnosed with symptomatic irreversible pulpitis.

## Introduction

Dental caries, being a persistent infectious ailment, poses a significant worldwide public health issue. The condition of untreated caries is widely prevalent, with a global prevalence rate of 34.1%, making it a significant focus of the comprehensive "Global Burden of Disease" assessment and it continues to be the primary cause of tooth loss [1]. Untreated dental caries can lead to endodontic diseases, which have a significant impact on the quality of life related to oral health, primarily manifesting as moderate physical pain caused by pulpitis [2]. According to a systematic review, the average prevalence of preoperative endodontic-associated pain was found to be as high as 81% [3]. Hence, the presence of endodontic pain can significantly impact an individual's overall quality of life and simultaneously emerge as the predominant self-reported factor contributing to tooth extraction [4]. Root canal therapy is typically the preferred treatment for permanent teeth exhibiting symptomatic irreversible pulpitis. Research findings have indicated a lack of significant correlation between the histological condition of the pulp and both clinical symptoms and diagnostic data [5,6]. Furthermore, it is possible to observe a well-preserved histological arrangement in the coronal and root pulp regions located a few millimeters away from the inflamed pulp [7]. The removal of pulp from the root results in a reduction in pulp volume, leading to the loss of the immune defense mechanism associated with the pulp [8]. According to reports, teeth exhibiting irreversible pulpitis possess the capacity for healing if effective management of pulpal inflammation is achieved [1]. Therefore, it is believed that a pulpotomy can serve as a viable alternative to root canal therapy in adult patients. Furthermore, within the realm of treatment methods for patients suffering from symptomatic irreversible pulpitis, pulpotomy has demonstrated superior efficacy in pain relief [8]. Pulpotomy refers to a surgical procedure that involves the extraction of the coronal pulp tissue with the aim of safeguarding the remaining radicular pulp [2]. The objective of this study is to promote the formation of dentin bridges through the utilization of different materials to encapsulate the residual pulp tissue, leveraging the dentinogenic capabilities of pulp cells. Calcium hydroxide is considered to be among the materials in question. Furthermore, Stanley advocated for the utilization of calcium hydroxide in vital pulp therapies [4]. The utilization of calcium hydroxide remains prevalent in contemporary vital pulp treatments, as it possesses notable attributes, including the ability to stimulate the formation of dentin bridges upon application to the exposed pulp surface. Previous research has examined the hemostatic and antibacterial properties of various solutions and laser techniques in the context of vital pulp treatments [8,9]. The utilization of lasers significantly reduces the potential for hemorrhaging, mechanical harm, and bacterial infection. Therefore, it has the potential to be efficacious in pulpotomy procedures [5]. According to available reports, the utilization of the potassium titanyl phosphate (KTP) laser at suitable parameters has been found to be devoid of any detrimental effects on pulp or periodontal tissues [6]. The KTP laser is believed to have a significant impact on mitigating acute postoperative pain, expediting the duration of bleeding control, and ensuring effective hemostasis [9]. Sodium hypochlorite (NaOCl) is a recommended solution for use in vital pulp treatments due to its hemostatic and antibacterial properties. According to reports, NaOCl has demonstrated biological compatibility with pulp tissues and has shown favorable outcomes as a hemostatic agent in vital pulp treatments [6].

## Materials and methods

This study is based on records of patients with various permanent molars diagnosed with symptomatic irreversible pulpitis treated by pulpotomy using NaOCl or KTP laser. This study was done after taking permission from the ethical committee (MRIDS/2022/EC27).

All treatment procedures were part of routine care, in which patients were verbally informed. The clinical records of a sample of 100 patients who satisfied the specified inclusion criteria were subjected to analysis. The analyzed dataset comprised various variables, namely, age, gender, presence of a percussion, tooth arch, electric pulp test, hemostasis time, presence of a periapical lesion, analgesic consumption postoperatively, and preoperative and postoperative pain scores. The selection criteria for patients undergoing pulpotomy with KTP laser and NaOCl were as follows: individuals aged 20 years or older, absence of any systemic disease, presence of a single tooth in each patient, indication of irreversible pulpitis with or without symptomatic apical periodontitis, positive response to electrical and thermal stimuli, restorability of the tooth, normal limits of probing pocket depth (3 mm), and mobility, and consistent treatment and follow-up by the same operator. The exclusion criteria encompassed the following factors: the existence of allergies, recent utilization of analgesics within the preceding 12-hour period, or administration of antibiotics within the last week prior to the procedure. The data from a cohort of 50 patients who received treatment with NaOCl and another cohort of 50 patients who received treatment with KTP laser were subjected to analysis. The selection of agents for dental application was conducted randomly.

Methodology

Articaine was utilized to achieve local anesthesia. Following the application of rubber-dam isolation, the tooth crown underwent disinfection using a 5% NaOCl solution. The cavity was prepared according to the principles of cavity preparation. The removal of necrotic and infected dentin was conducted by employing an excavator and/or a round bur in a low-speed handpiece. The cavity was thoroughly cleansed using a cotton pellet that had been saturated with a 5% NaOCl solution. Subsequently, the pulp chamber was exposed by carefully removing the roof using a sterile bur. The coronal pulp tissue was extracted using a sterile round bur attached to a water-cooled high-speed handpiece. The initial hemorrhage was managed by applying a cotton pellet saturated with physiological saline. The achievement of complete hemostasis and cavity disinfection was accomplished through the utilization of either a 2.5% NaOCl solution or a KTP laser, depending on the respective experimental groups.

In group A, hemostasis and cavity disinfection were done using NaOCl. In this experimental cohort, the attainment of hemostasis was accomplished through the examination of a cotton pellet saturated with a 2.5% NaOCl solution, which was positioned within the pulp chamber. The cotton pellet was assessed at two-minute intervals until the six-minute mark, and the duration of hemostasis was duly documented. In addition, the NaOCl served as the disinfectant for the cavity.

Group B focuses on the application of KTP laser for hemostasis and cavity disinfection. In this study, the attainment of comprehensive hemostasis and cavity disinfection was accomplished through the utilization of a KTP laser operating at a wavelength of 532 nm. The laser was operated at a power of 1.5 W, employing both pulse and non-contact modes, with a ton of 100 ms and toff of 100 ms, for a duration of two seconds. The optical fiber possessed a diameter of 300 µm. The laser application was iterated up to three times if deemed necessary. Following the successful achievement of bleeding control, the process of cavity disinfection was carried out through the application of laser technology. The laser used had a power output of 1 watt and was equipped with a 300 µm tip. The disinfection procedure involved employing a circular motion technique in a non-contact mode for a duration of five seconds. Subsequently, calcium hydroxide was applied to the pulp orifices and the floor of the pulp chamber. The application of glass-ionomer cement was performed subsequent to the placement of calcium hydroxide. The tooth was subjected to permanent restoration using composite resin during the same session. The patients were administered a combination of 400 mg ibuprofen and 500 mg acetaminophen every six hours during the initial two-day period following surgery to manage intense postoperative pain.

The patients were provided with a questionnaire to evaluate pain levels before and after surgery. The patients documented their levels of postoperative pain at specific time intervals, including the sixth hour, first day, second day, third day, and eighth day. This was done using a 100 mm visual analog pain scale, where a marking of 0 mm indicated no pain and a marking of 100 mm indicated the highest level of pain, reflecting the severity of the pain experienced.

Statistical analysis

The statistical analysis was conducted using the IBM SPSS 22.0 software (IBM Corp., Armonk, NY), which is commonly referred to as the Statistical Package for Social Sciences. The normality of the data was assessed using the Kolmogorov-Smirnov test. The statistical analysis of the data was conducted using the Mann-Whitney U, Friedman, and chi-square tests, with a significance level set at P < 0.05.

## Results

Table 1 presents an analysis of the basic profile and clinical data. There were no statistically significant differences observed between the two groups with respect to age, gender, periapical status (radiographic), percussion test, preoperative electric pulp test, duration of hemostasis, and location of the tooth (P > 0.05). The pain score of group B was significantly lower than that of group A on the first day (P < 0.05). While no notable disparity was detected among the groups during the remaining postoperative periods. It is worth noting that the KTP laser exhibited comparatively lower pain scores (P > 0.05). In both groups, the initial pain score before surgery was found to be significantly higher than the pain scores recorded at all subsequent time intervals after surgery (P < 0.05). Within group A, it was observed that the pain score during the sixth hour after the surgical procedure was notably greater compared to the pain scores recorded during all other time intervals following the operation (P < 0.05). In group B, the pain score at the sixth hour exhibited a statistically significant increase compared to the pain scores observed on the third day and eighth day (P < 0.05; Table 2).

**Table 1:** Basic profile of the patients in groups A and B

Basic profile	Group A		Group B		P
Gender	Number	Percentage	Number	Percentage	
Male	30	60	33	66	0.19
Female	20	40	17	34	
Age	26.11 ± 5.15		27.19 ± 5.19		0.22
Duration of hemostasis	3.58 ± 0.98		2.83 ± 0.87		0.33
Preoperative electric pulp test	28.09 ± 4.55		31.15 ± 4.99		0.11
Periapical status/radiographic					
Normal	34	68	33	66	0.46
Radiolucency or widening of periodontal ligament	16	32	17	34	
Pre and intraoperative factors					
Percussion test					
Positive response	45	90	47	94	0.43
Location of the tooth					
Maxillary	18	36	10	20	0.45
Mandibular	32	64	40	80	

**Table 2:** Pain rating scores in groups A and B

	Group A	Group B	P
Preoperative pain	71.11 ± 4.69	67.52 ± 4.11	0.15
Postoperative 6^th^ hour	26.98 ± 3.19	21.55 ± 3.14	0.06
Postoperative 1^st^ day	14.11 ± 2.15	9.14 ± 2.11	0.004
Postoperative 2^nd^ day	12.02 ± 1.19	7.14 ± 1.11	0.21
Postoperative 3^rd^ day	8.88 ± 1.08	2.41 ± 0.92	0.34
Postoperative 8^th^ day	5.22 ± 0.88	0.79 ± 0.09	0.36

## Discussion

The field of pain research has experienced a gradual increase in its significance within the realms of medical and dental sciences with pain management during both the intraoperative and postoperative phases becoming a very crucial element in the field of endodontology [1]. Despite recent advancements in the field of endodontology, a significant proportion of patients, approximately 58%, continue to report experiencing pain following endodontic procedures [6]. Postoperative endodontic pain is a complex phenomenon influenced by multiple factors. Previous studies have endeavored to establish a correlation between it and predictive factors, including determinants related to the patient and tooth, as well as pre- and intraoperative conditions [1]. Nevertheless, the outcomes have frequently exhibited a degree of uncertainty, unpredictability, or a dearth of apparent causal connections. In their study, Galani et al. [5] conducted a comparison of postoperative pain levels and success rates between pulpotomy and root canal treatment procedures. The study findings indicate that the pulpotomy group experienced reduced levels of postoperative pain and discomfort. Furthermore, there have been reports indicating that pulpotomy treatment results in a nearly 50% reduction in treatment duration when compared to root canal treatment [7]. The pulpotomy procedure has the potential to enhance patient comfort due to its relatively shorter duration. This retrospective study aimed to assess the clinical outcomes of NaOCl and KTP laser, both commonly employed for their hemostatic and disinfection properties, in the management of postoperative pain following pulpotomy treatment in permanent teeth exhibiting symptomatic irreversible pulpitis. The existing body of literature on the assessment of post-pulpotomy pain in permanent teeth is relatively scarce [5,7].

The current discourse among researchers revolves around the diagnostic nomenclature pertaining to reversible and irreversible pulpitis. The determination of irreversible pulpitis relies on clinical diagnostic procedures rather than histological findings. However, it is worth noting that these clinical diagnostic procedures may exhibit limitations in terms of reliability. The study conducted by Ricucci et al. [8] aimed to assess the dependability of clinical diagnosis in distinguishing between reversible and irreversible pulpitis, in comparison to histological diagnosis. Based on the findings of the study, there was an observed consistency rate of 84.4% between the clinical and histological manifestations of irreversible pulpitis. Moreover, various studies have documented that the preservation of the healthy pulp following the removal of the inflamed pulp can be achieved with a significantly high rate of success [9,10]. The process of wound healing is a pathological phenomenon that operates on a similar fundamental principle in the pulp as it does in other bodily organs. The present investigation incorporated and examined data from patients diagnosed with irreversible pulpitis, as indicated by the aforementioned studies. The current study revealed a notable decrease in pain values during the first week in comparison to the preoperative level.

Calcium hydroxide is widely regarded as a highly advantageous pulp-capping agent [11]. Calcium hydroxide exhibits a significantly elevated pH level, thereby promoting the activation of fibroblasts and enzyme systems. Additionally, it effectively counteracts the acidic nature of low pH substances, possesses antibacterial characteristics, and facilitates the protection and restoration of dental pulp [12]. The primary rationale for employing calcium hydroxide as a pulp-capping agent lies in its inherent antibacterial properties [11,12]. According to the literature, calcium hydroxide has been identified as a highly suitable substance for the purpose of pulp protection and as a biocompatible agent. Nevertheless, its compressive strength is significantly low [11,12]. Undesirable characteristics associated with calcium hydroxide include inflammation and necrosis occurring on the surface of the pulp, toxic effects on cells, the presence of tunnel defects in newly-formed tertiary dentin bridges, and dissolution when used under permanent restorations. Numerous studies have indicated that mineral trioxide aggregate (MTA) exhibits greater efficacy compared to calcium hydroxide when used as a pulp-capping agent [13-15]. Nevertheless, the use of MTA is not without its drawbacks, including challenges in its handling, extended setting time, and elevated cost. Several studies have compared the efficacy of calcium hydroxide and MTA in relation to pain management and long-term outcomes [16,17]. In a previous study, the superiority of MTA was established [17]. However, another previous study reported no discernible difference between MTA and calcium hydroxide. In the present studies, calcium hydroxide was employed as a pulp-capping agent [16]. In the present study, as well as in numerous other studies, NaOCl was employed as a hemostatic agent [5,17]. Hafez et al. underscored the significance of achieving hemostasis with NaOCl in conjunction with achieving a hermetic closure [18]. In addition to its notable hemostatic capabilities, NaOCl exhibits antibacterial properties; it effectively disinfects dentin that is contaminated near the site of exposure and serves as a preventive measure against fibrin formation. According to previous research findings by Witton et al., prolonged exposure to NaOCl in the alveolar bone from root canal procedures has been linked to neurological damage [19,20]. The reduction of pain may be facilitated by the impact of NaOCl application on neurogenic sensors, as suggested by previous research [19]. In their study, Ballal et al. evaluated the impact of two different solutions, namely, a 2.5% NaOCl and physiological saline, on the pulpal wound cleansing process [20]. The teeth under investigation were asymptomatic and exhibited deep caries. The use of NaOCl solution has been documented to result in a substantial decrease in postoperative discomfort and early painful failures when compared to physiological saline. In the current investigation, it was observed that NaOCl exhibited elevated pain scores across all time intervals in comparison to KTP laser. However, statistical significance was only observed at the 24-hour mark.

The utilization of laser technology in surgical procedures is experiencing a steady rise due to its notable attributes, including prompt hemostasis, diminished risk of contamination, establishment of a sterile surgical environment, and alleviation of postoperative edema and pain [21]. The KTP laser is a type of laser that utilizes frequency doubling of a neodymium-doped yttrium aluminum garnet (Nd:YAG) laser, resulting in a wavelength of 532 nm. This specific wavelength has the ability to be absorbed in a selective manner by oxyhemoglobin. In a study conducted by Liu, an evaluation was performed to assess the effects of Nd:YAG laser pulpotomy and formocresol pulpotomy on primary teeth in humans [22]. The findings indicated that the success rate of Nd:YAG laser pulpotomy was significantly superior to that of formocresol pulpotomy. The study conducted by Fornaini et al. examined the utilization of low-power (1 watt in continuous wave) laser-assisted KTP laser surgery, focusing on the intervention characteristics and patient compliance [23]. According to the literature, the low-power KTP laser has been found to offer effective pain management and facilitate the process of recovery. In a study conducted by Auf et al., a comparison was made between KTP laser tonsillectomy, utilizing a power range of 12-14 W in cutting mode, and the conventional standard tonsillectomy procedure [24]. According to the report, the KTP laser group exhibited reduced blood loss. Additionally, while initial pain levels were lower (up to day one), the wound in this group experienced greater long-term discomfort as a result of delayed healing. The current study observed that the average pain scores of the KTP laser group were consistently lower across all time intervals. The occurrence of photochemical effects that lead to biostimulation and pain inhibition in response to low power density has been observed [23]. The potential rationale behind the efficacy of the low-power KTP laser in managing postoperative pain could be elucidated by this statement. According to reports, the utilization of the KTP laser at elevated power levels has been associated with the potential occurrence of secondary infections. This is attributed to the photothermal effects induced by the laser, including evaporation, carbonization, burning, coagulation, and hyperthermia within the tissues. The occurrence of secondary infections can potentially result in the postponement of wound healing and the experience of pain [23]. Furthermore, the potential placebo effect associated with laser treatment may play a role in the reduction of postoperative pain. The observed phenomenon encompasses an intricate combination of physiological and psychological interactions.

The perception of pain is a subjective and variable experience that is influenced by both physical and psychological factors. The precise and dependable evaluation of pain holds significant importance in the realm of pain research and the implementation of effective pain management strategies. Various pain scales, such as the visual analog scale (VAS), have been employed in numerous research studies for the purpose of evaluating the intensity of pain. The VAS demonstrates satisfactory validity and reliability as a tool for assessing the intensity of pain and discomfort experienced by individuals, given that it is appropriately designed and administered [25].

The findings of this study indicate a decrease in mean pain scores during the post-pulpotomy periods. The aforementioned findings align with results obtained from other clinical studies [5], which demonstrate a notable decrease in pain subsequent to pulpotomy treatment. The findings from these studies underscore the efficacy of pulpotomy as a means of alleviating pain. The severing of the distal portions of nociceptive sensory neurons during pulpotomy treatment has the potential to impact the intensity of postoperative pain by reducing local tissue pressure and concentrations of inflammatory mediators [6].

## Conclusions

The KTP laser group exhibited a lower postoperative pain score in comparison to the NaOCl group. The utilization of KTP laser-assisted pulpotomy demonstrated enhanced efficacy in alleviating pain among individuals diagnosed with symptomatic irreversible pulpitis.
